# Use of OCT Imaging in the Treatment of Right Coronary Artery Occlusion Causing NSTEMI as an Initial Complication of Essential Thrombocythaemia

**DOI:** 10.1155/cric/6647538

**Published:** 2026-02-25

**Authors:** Robert Michael, Jith Somaratne, Nicola Eaddy

**Affiliations:** ^1^ Department of Cardiology, Auckland City Hospital, Auckland, New Zealand, adhb.govt.nz; ^2^ Department of Haematology, Auckland City Hospital, Auckland, New Zealand, adhb.govt.nz

**Keywords:** acute myocardial infarction, angiography, essential thrombocythaemia, NSTEMI, OCT, optical coherence tomograph

## Abstract

**Background:**

Essential thrombocythaemia (ET) is a chronic myeloproliferative neoplasm characterised by clonal thrombocytosis and an overall increased risk of thrombotic events. Acute coronary syndrome as an initial presentation of ET is rare in the literature.

**Case Summary:**

A 39 year old woman with established ET presented with non‐ST‐elevation myocardial infarction (NSTEMI). Coronary angiography revealed a hazy, eccentric mid‐right coronary artery lesion. Optical coherence tomography (OCT) demonstrated thrombus without atherosclerotic plaque. OCT‐guided direct stenting achieved optimal expansion with thrombolysis in myocardial infarction (TIMI) Grade III flow. She was discharged on dual antiplatelet therapy and referred to haematology for ET management. At three months, she remained asymptomatic on clinic follow‐up.

**Conclusion:**

OCT provided diagnostic and procedural guidance in a rare thrombotic NSTEMI secondary to ET. This case highlights the role of intracoronary imaging in differentiating thrombosis from atherosclerotic plaque rupture.

## 1. Introduction

Essential thrombocythaemia (ET) is a myeloproliferative neoplasm characterised by an increased platelet count >450 × 10^9^/L and megakaryocytic hyperplasia [1]. It frequently involves the mutations JAK2, V617F, MPL or CALR [2, 3] of which the presence confers an independent increased thrombotic risk. Although thrombotic events are common, myocardial infarction is an infrequent initial manifestation of ET (up to 10% over long‐term follow‐up case series) [4–6]. No randomised trials exist to guide acute coronary syndrome (ACS) management in ET, particularly revascularisation or antiplatelet strategy. This case demonstrates the use of optical coherence tomography (OCT) guided PCI for thrombus‐mediated NSTEMI occurring with ET. [7–12].

## 2. Patient Information

A 39 year old female presented to hospital with two weeks of crescendo exertional chest pain. She had been diagnosed with ET nine years earlier during a prior pregnancy. She was not on regular cytotoxic medical therapy. Her platelet count three months prior was recorded as 760 × 10^9^/L. Otherwise she had a background of untreated hypertriglyceridaemia, one prior miscarriage and regular oral contraceptive use. She was a never smoker with no family history of premature coronary disease.

### 2.1. Clinical Findings

On examination, she had normal vesicular breath sounds and dual heart sounds with no murmur. Jugular venous pressure was not elevated and there was no peripheral oedema present. Her heart rate was 76 beats per minute with a blood pressure of 157/74 mmHg.

### 2.2. Timeline (See Table 1 Below)

**Table 1:** Timeline of clinical events.

**Event**	**Timing**
Exertional chest pain onset	2 weeks preadmission
Crescendo pattern	3 days preadmission
Hospital admission and angiography	Day 0
OCT‐guided PCI	Day 0
Echocardiography	Day 1
Discharge	Day 2
Follow‐up	3 months asymptomatic

### 2.3. Diagnostic Assessment

**Figure A1:**
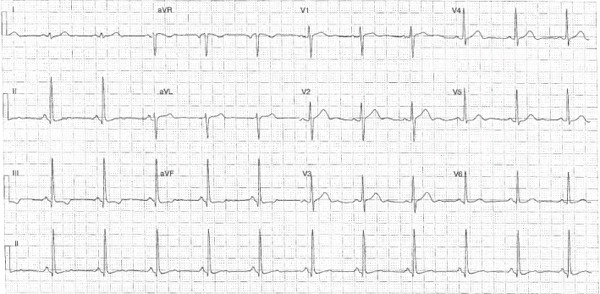
12 Lead ECG on index admission (preintervention).

**Figure A2:**
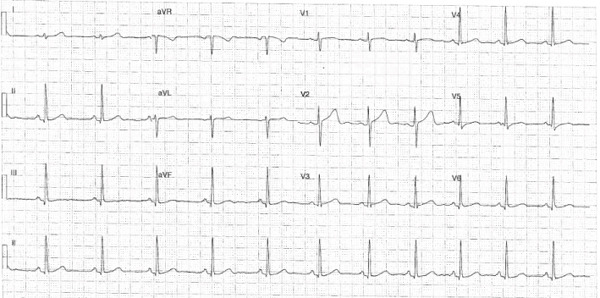
12 Lead ECG on index admission (post intervention).

A 12‐lead electrocardiogram (ECG) on admission demonstrated inferior ischaemic changes with 1 mm of ST‐segment depression in leads II and aVF (Figures A1 and A2). These changes were not observed post PCI (Figure A2). The peak high‐sensitivity troponin T (hsTnT) was 35 ng/L (upper limit of normal 15 mg/L) on day two.

A routine full blood count on admission revealed a platelet count of 724 × 10^9^/L and haemoglobin of 149 g/L. Her other laboratory tests were unremarkable (Table 2).

**Table 2:** Laboratory test table.

**Investigation (unit)**	**Value**
Hb (115–155) g/L	149
RBC (3.6–5.6) E + 12/L	4.77
WBC (4–11) E + 9/L	7.55
Platelets (150–400) E+9/L	724
Troponin T (< 15) ng/L	32
Na (135–145) mmol/L	138
K (3.5–5.2) mmol/L	3.7
Urea (3.2–7.7) mmol/L	3.7
Creatinine (45–90) *μ*mol/L	61
Cholesterol (< 5) mmol/L	4.2
LDL(< 3.4) mmol/L	2.2
Triglycerides (< 2) mmol/L	0.8
HDL (> 1) mmol/L	2.9

The Global Registry of Acute Coronary Events (GRACE) score was calculated at 83 (0.5%).

A diagnosis of non‐ST‐elevation myocardial infarction (NSTEMI) was made and subcutaneous enoxaparin was administered alongside dual antiplatelet therapy (DAPT) in the form of aspirin and ticagrelor.

### 2.4. Therapeutic Intervention

**Figure A3:**
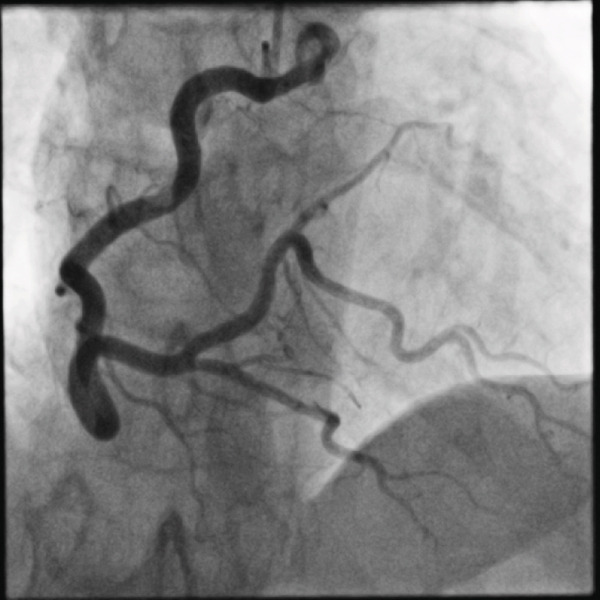
Invasive coronary angiogram (preintervention).

**Figure A4:**
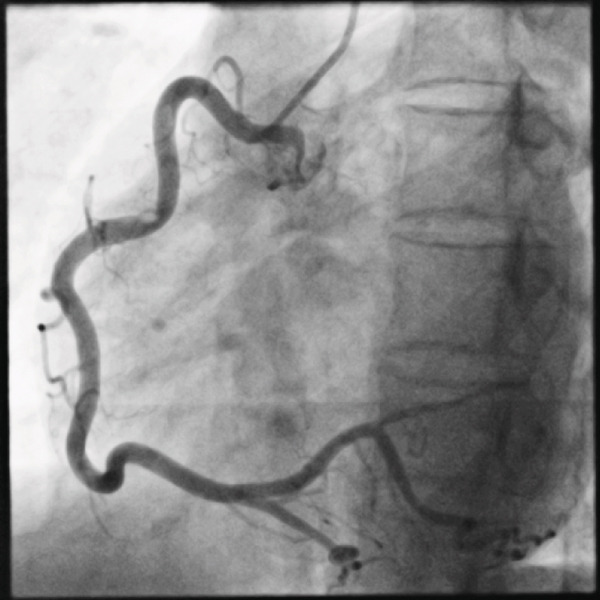
Invasive coronary angiogram (preintervention).

**Figure A5:**
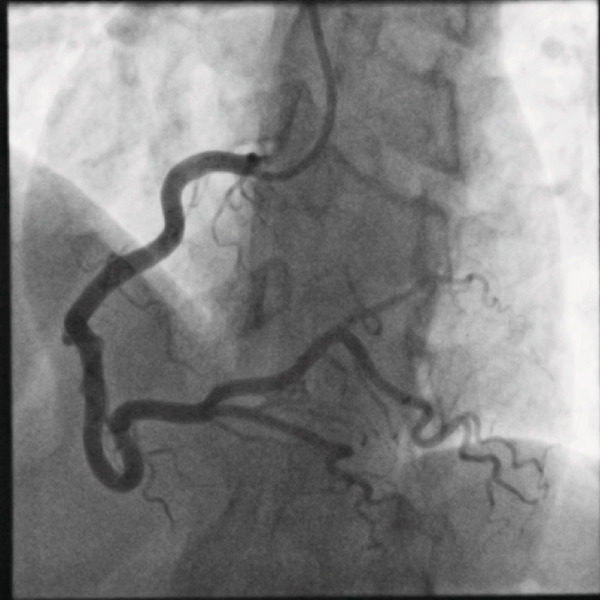
Invasive coronary angiogram (post intervention).

**Figure A6:**
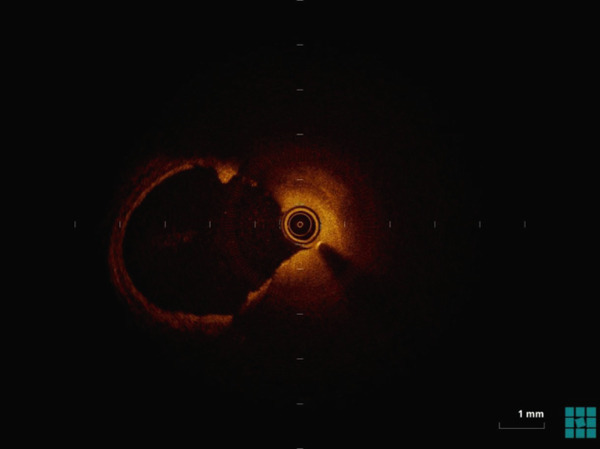
OCT Figure A1 (preintervention).

**Figure A7:**
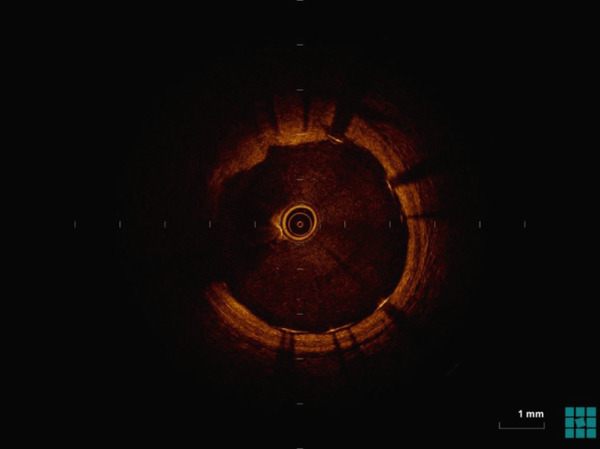
OCT Figure A2 (postintervention).

Invasive coronary angiography was undertaken via a 6F right radial artery access using a Radifocus Optitorque 5F Tiger catheter (Terumo Corporation, Tokyo, Japan). It showed no evidence of disease in the left coronary system. There was a hazy, eccentric 70% stenosis in the mid‐right coronary artery (Figures A3 and A4). A SION blue (Asahi Intecc, Aichi, Japan) guidewire was placed in the distal vessel via a 6F Amplatz Left 1 guiding catheter. OCT was undertaken using a Dragonfly OPTIS imaging catheter (Abbott Vascular, Santa Clara, California, United States). This demonstrated normal vessel morphology throughout. At the site of the angiographic stenosis, there was no atherosclerotic plaque but there was substantial thrombus (Figure A6). A strategy of direct stenting was used with deployment of a Resolute Onyx 4.5 × 15 mm (Medtronic, Santa Rosa, California, United States) drug‐eluting stent at nominal pressure. Subsequently, the stent was postdilated with a 5.0 × 8 mm NC Emerge balloon (Boston Scientific Corporation, Marlborough, Massachusetts, United States). An excellent final angiographic result was obtained with thrombolysis in myocardial infarction (TIMI) Grade III flow and no evidence of distal embolisation (Figure A5). The post‐PCI OCT imaging confirmed excellent stent expansion and apposition (Figure A7).

### 2.5. Follow‐Up and Outcomes

Transthoracic echocardiography on the second day of the index admission demonstrated normal left ventricular size and ejection fraction (60%–65%) with no regional wall motion abnormalities. Right heart size and function were also normal. There were no significant valvular abnormalities.

The patient was discharged on day two of her admission with a trivial decrease in hsTnT (33 ng/L). A 12 month course of uninterrupted DAPT was recommended. At outpatient clinic follow‐up three months post discharge, she was well with no ongoing symptoms or sequelae related to her NSTEMI. Repeat platelet count at three months was 410 × 10^9^/L.

## 3. Discussion

Essential thrombocythaemia increases thrombotic risk via megakaryocytic proliferation, JAK2‐mediated cytokine activation and platelet hyperreactivity [1, 2, 13].

Overall young patients with ET presenting with chest pain must be carefully evaluated for obstructive coronary artery disease. Often ET is first suspected with an absolute increase in the platelet count on initial workup.

Furthermore, once suspected, both hypertension and cigarette smoking have been described as additional risk factors for thrombosis development [13]. Females have a higher predisposition for thrombosis, and there is case‐series evidence of a higher burden of myocardial infarction in the literature to date.

The optimal treatment strategy for patients with ET and ACS is not clear: medical therapy with antithrombotic agents, PCI and CABG have all been described in the literature. In the acute setting, the selection of strategy is primarily based on the presence of ongoing myocardial ischaemia. Continued cytoreductive therapy (hydroxyurea or anagrelide) and long‐term antiplatelet therapy often reduces recurrence risk [14–17].

The use of OCT offers superior resolution, helping distinguish plaque morphology and ensures stent expansion in prothrombotic states. In particular, OCT confirmed thrombus without plaque rupture, guiding safe direct stenting and post‐PCI optimisation [9, 18].

To our knowledge, this case highlights one of the few reports of ET in the literature that intracoronary OCT was used. In particular to guide choice of treatment strategy of NSTEMI that included drug eluting stent placement. In our case, perhaps early initiation of both antiplatelet and cytotoxic drugs and stenting with confirmation of stent apposition may have reduced the risk of further short term complications.

However we acknowledge that there is insufficient published data to guide the management of ET patients presenting with NSTEMI and therefore is a need for further study.

## 4. Conclusion

Optical coherence tomography provide additional diagnostic and procedural information in this case of thrombotic NSTEMI due to ET. Integration of intracoronary imaging and may have a role in acute management decisions, but further research is needed.

## Ethics Statement

This case report was conducted in accordance with the ethical standards of the institutional and national research committees and with the 1964 Helsinki Declaration and its later amendments. Formal ethics approval was not required as this study describes a single clinical case with no identifiable patient data.

## Consent

Written informed consent was obtained for publication of clinical details and images.

## Patient Perspective

The patient was relieved at rapid symptom resolution as an inpatient and understood the need for combined cardiology and haematology follow‐up. She is doing well on three month follow‐up with absence of symptoms.

## Disclosure

All authors approved the final version for submission.

## Conflicts of Interest

The authors declare no conflicts of interest.

## Author Contributions

Robert Michael: Involved in the conceptualisation, investigation, writing, original draft, visualisation, and project administration. Jith Somaratne: supervision, validation, writing, review and editing, and overall cardiology clinical oversight. Nicola Eaddy: supervision, validation, writing, review and editing and overall haematology oversight. All authors contributed to the clinical care, diagnosis, and management of the patient and participated in the drafting and revision of the manuscript.

## Funding

No funding was received for this manuscript.

## Supporting Information

Additional supporting information can be found online in the Supporting Information section.

## Supporting information


**Supporting information** CARE checklist—case reports in cardiology.

## Data Availability

The data that support the findings of this study are available on request from the corresponding author. The data are not publicly available due to privacy or ethical restrictions.
