# Supplementary material for: Use of OCT Imaging in the Treatment of Right Coronary Artery Occlusion Causing NSTEMI as an Initial Complication of Essential Thrombocythaemia
Source: Case Rep Cardiol. 2026 Feb 25;2026:6647538. doi: 10.1155/cric/6647538 (PMC12933134; doi:10.1155/cric/6647538)
Supplement: Supplementary file 1 — Supporting information CARE checklist—case reports in cardiology. [file CRIC-2026-6647538-s001.docx]

# CARE Checklist – Case Reports in Cardiology

Case: Use of OCT imaging in the treatment of right coronary artery occlusion causing NSTEMI as an initial complication of essential thrombocythaemia

| **Section** | **Description** |
| --- | --- |
| Title | Identifies the report as a case report; includes the key condition (essential thrombocythaemia), intervention (OCT-guided PCI/stenting), and outcome (NSTEMI). |
| Key Words | NSTEMI, Essential thrombocythaemia, OCT, Acute myocardial infarction, Optical coherence tomography, Angiography. |
| Abstract | Structured summary highlighting the case, rarity of Essential thrombocythaemia as an initial ACS presentation, and OCT-guided intervention to help facilitate decision making and treatment strategy. |
| Introduction | Describes Essential thrombocythaemia background, epidemiology, genetic mutations (JAK2/MPL/CALR), thrombotic complications, and rationale for reporting. |
| Patient Information | 39-year-old female with Essential thrombocythaemia, oral contraceptive use, platelet count 724×10⁹/L, prior hydroxyurea therapy stopped due to pregnancy. |
| Clinical Findings | Crescendo angina, retrosternal chest pain, inferior ischaemic ECG changes, elevated hs-troponin T. |
| Timeline | Two weeks of chest pain worsening over three days before presentation; PCI performed on admission; discharged day 2; followup at 3 months. |
| Diagnostic Assessment | Coronary angiography showing mid-RCA thrombus; OCT confirming thrombus without plaque rupture; echo showing normal LV function. |
| Therapeutic Intervention | OCT-guided PCI and post-dilatation; DAPT (aspirin + ticagrelor) and enoxaparin; haematology follow-up. |
| Follow-up and Outcomes | Discharged day 2; asymptomatic at 3 months; platelet counts monitored; no recurrence of ischaemia. |
| Discussion | Reviews literature on ET-related coronary thrombosis; management options (DAPT, thrombectomy, stenting); OCT vs IVUS utility; cytoreductive therapy discussion. |
| Patient Perspective | The patient expressed satisfaction with rapid recovery and understood importance of continued follow-up. |
| Informed Consent | Written informed consent for publication obtained from the patient. |
| Ethical Approval | Not required for a single anonymised case report; conducted per institutional and Helsinki principles. |
| Conclusion | Summarises novelty of OCT-guided PCI in ET-associated NSTEMI and calls for future research into management. |
| References | Formatted in Vancouver style as per journal requirements. |
| Acknowledgements / Funding / Conflicts | No funding received; no conflicts declared; authors contributed per CRediT taxonomy. |
